# A Review of the Pathological and Molecular Diagnosis of Primary Myelofibrosis

**DOI:** 10.3390/cancers18010050

**Published:** 2025-12-24

**Authors:** Richard Shao, Christopher Ryder, Le Wang, Hailing Zhang, Lynn Moscinski, Michael Martin, Mac Shebes, Julie Y. Li, Jinming Song

**Affiliations:** 1 College of Medicine, University of Central Florida, 6850 Lake Nona Blvd, Orlando, FL 32827, USA; 2 Department of Pathology, H. Lee Moffitt Cancer Center and Research Institute, Tampa, FL 33612, USA; 3 Reading Hospital McGlinn Cancer Institute, Phoenixville Hospital Campus, West Reading, PA 19460, USA; 4 College of Medicine, University of South Florida Morsani, Tampa, FL 33612, USA; michaelseanmartin@usf.edu (M.M.); macs@usf.edu (M.S.)

**Keywords:** primary myelofibrosis, PMF, JAK2, CALR, MPL, pathogenesis, molecular, diagnosis

## Abstract

Primary myelofibrosis (PMF) is a Philadelphia chromosome (Ph)-negative myeloproliferative neoplasm (MPN) that features clonal proliferation of atypical megakaryocytes and myeloid cells, fibrosis of the bone marrow, extramedullary hematopoiesis, and increased risk of leukemic transformation to acute myeloid leukemia (AML). With the widespread application of molecular studies, especially next generation sequencing (NGS), significant advances have reshaped our understanding of the molecular pathogenesis of PMF and the prognostic relevance of specific gene mutations. In this review, we summarize its clinicopathologic features, genetic and molecular findings, updated diagnostic criteria, and differential diagnosis. These updates have been incorporated into the 5th edition of the World Health Organization classification of Hematolymphoid Tumors (WHO-5th) and the 2022 International Consensus Classification (ICC), thereby improving diagnostic accuracy and risk stratification, both of which are essential for tailoring treatment strategies and enhancing patient outcomes.

## Simple Summary

Primary myelofibrosis (PMF) is a myeloproliferative neoplasm (MPN) that features clonal proliferation of atypical megakaryocytes and myeloid cells, fibrosis of the bone marrow, and increased risk of leukemic transformation to acute myeloid leukemia (AML). In this review, we summarize its clinicopathologic features, genetic and molecular findings, updated diagnostic criteria, and differential diagnosis, in an aim to improve diagnostic accuracy and risk stratification, which are essential for tailoring treatment strategies and enhancing patient outcomes.

## 1. Introduction

Primary myelofibrosis (PMF) is a clonal hematopoietic stem cell malignancy characterized by abnormal proliferation of megakaryocytic and granulocytic lineages accompanied by progressive bone marrow fibrosis, osteosclerosis and extramedullary hematopoiesis. Its pathogenesis results from constitutive Janus kinase/signal transducers and activators of transcription (JAK/STAT) signaling, especially driven by mutations in *JAK2*, *MPL*, or *CALR*, leading to cytokine overproduction and the development of a fibrotic bone marrow microenvironment [1].

PMF carries a high risk of transformation to AML (~20%), compared to other myeloproliferative neoplasms such as essential thrombocythemia (ET, ~1%) and polycythemia vera (PV, ~4%) [2]. The overall median survival time in PMF is 9.2 years, with 15- and 20-year survival rates of 32% and 20% [3]. The principal causes of death include infection, thrombosis, cardiac or pulmonary failure, and transformation to AML [4].

There are significant overlaps in the clinical and morphologic features between PMF and other myeloproliferative neoplasms, particularly ET and PV. WHO-5th and ICC followed the proven classification scheme of the previous WHO classification that integrated morphological, clinical, cytogenetic and molecular features for diagnosis [5,6]. Our understanding of PMF has evolved remarkably through technologies like NGS and single-cell sequencing. This review provides a comprehensive overview of the clinicopathologic and molecular features of PMF, with additional detail on differential diagnosis and prognostic molecular markers.

## 2. Pathogenesis and Molecular Findings of Primary Myelofibrosis

Somatic mutations in PMF and other MPNs are categorized as “driver” mutations, such as *JAK2*, *MPL*, and *CALR*, and “non-driver” mutations, including *ASXL1*, *SRSF2*, *U2AF1*, *EZH2*, *IDH1*, *IDH2*, and others. In general, driver mutations are essential for establishing the MPN phenotype, whereas non-driver mutations contribute to disease progression and leukemic transformation [7]. *JAK2*, *CALR*, and *MPL* mutations are usually mutually but not absolutely exclusive, occurring in ~50–60%, 30%, and 5–10% of PMF cases, respectively [8]. *JAK2* mutation leads to downstream activation of signal transduction pathways including STAT, MAPK, PI3K, and Akt with subsequent activation of proliferation and anti-apoptotic genes, which lead to myeloproliferation and cytokine secretion [9]. It has been shown that low *JAK2*^V617F^ allele burden in PMF is associated with a more aggressive clinical course and inferior overall with leukemia-free survival [10]. It was speculated that these patients with low JAK2 V617F allele burden might have a more dominant JAK2 V617F negative clone with higher propensity to undergo clonal evolution, which is supported by increasing evidence that points to JAK2 V617F mutation as a secondary event in the clonal hierarchy of MPNs.

*MPL* encodes the thrombopoietin receptor (TPOR), and *MPL* mutants cause the receptor to adopt a constitutively active conformation independent of thrombopoietin binding. Mutant TPOR forms a stable dimer that activates the associated JAK2 kinase, triggering downstream JAK-STAT signaling, with resultant hematopoietic stem cell self-renewal and megakaryopoiesis [11]. *MPL* W515L, and W515K mutations (*MPL*W515L/K) are the most common *MPL* exon 10 mutations.

Mutant calreticulin (CALR) acquires an abnormal C-terminal sequence that allows it to bind TPOR in the endoplasmic reticulum. This aberrant interaction promotes ligand-independent activation of TPOR [12,13]. *CALR* mutations are divided into type 1 (52-bp deletion) and type 2 (5-bp insertion) mutations [14]. The type 1 mutations eliminate all negatively charged amino acids in the CALR C terminus, and the type 2 mutations eliminate about half of the negatively charged amino acids. Patients with type 1-like *CALR* mutations have significantly improved OS compared with patients with type 2-like mutations patients [15,16]. Compared with *JAK2* or *MPL* mutations, *CALR* mutation is associated with a decreased risk of thrombosis and longer overall survival in PMF, while there is no significant difference between *JAK2* or *MPL* mutations [13]. Table 1 summarizes the major morphologic and molecular features of major MPNs.

Approximately 5% of cases of PMF are “triple negative” (TN-PMF) without known driver mutations in *JAK2*, *CALR*, or *MPL* mutations [17]. In the absence of these three major driver mutations, screening for other mutations associated with myeloid neoplasms, such as *ASXL1*, *EZH2*, *TET2*, *IDH1*, *IDH2*, *SRSF2*, and *SF3B1*, can help establish the clonal nature of the disease [5]. TN-PMF portends a poor prognosis. Patients with TN-PMF more likely have thrombocytopenia and less frequent splenomegaly. TN-PMF tends to show increased incidence of trisomy 8 and more frequent *ASXL1/SRSF2* co-mutations than conventional PMF [17]. One study observed significantly decreased survival and more aggressive clinical behavior with higher rates of leukemic transformation and shorter duration of response to ruxolitinib [18]. Mutations affecting RNA splicing, epigenetic modification, and signaling (*SRSF2*, *SETBP1*, *IDH2*, *CBL*, and *GNAS*) are more common, which likely drive its aggressive course and may account for suboptimal responses to JAK inhibition [18]. TN-PMF diagnosis is challenging and requires exclusion of mimicking conditions such as MDS with fibrosis, secondary MF (post-ET/post-PV), CMML, CML (BCR-ABL1+), and essential thrombocythemia, as well as acute leukemias and marrow metastases. A comprehensive workup—including bone marrow biopsy, cytogenetics, molecular testing, and immunohistochemistry—is essential.

Additional “non-driver” mutations, mostly affecting genes of epigenetic modifiers or spliceosome components, such as *ASXL1* (21.7%), *TET2* (9.7%), *SRSF2* (8.5%), *DNMT3A* (5.7%), *EZH2* (5.1%), *CBL* (4.4%), and *IDH1/2* (2.6%) are frequently identified in PMF by NGS studies and have been incorporated into prognostic models [19,20]. Of these, mutations in *ASXL1*, *EZH2*, *SRSF2,* and *IDH* are associated with increased risk of premature death or leukemic transformation [19]. Accordingly, high-molecular risk (HMR) mutations are defined as mutations in *ASXL1*, *SRSF2*, *EZH2*, *IDH1*, and *IDH2*. *TP53* and *U2AF1* mutations have been included in the HMR category in some prognostic models [7]. The presence of ≥1 HMR mutations is independently associated with inferior overall survival (OS) while leukemia-free survival (LFS), and ≥2 HMR mutations are associated with a dismal outcome [21]. In a cohort of 363 PMF patients, Loscocco et al. assessed the prognostic impact of additional mutations (*CBL*, *NRAS*, *KRAS*, *RUNX1*, *TP53*). Univariate analysis showed that *CBL*, *NRAS*, *KRAS*, and *TP53* mutations were significantly associated with inferior overall survival, while *RUNX1* had borderline significance. However, in multivariate models that included HMR mutations (*ASXL1*, *SRSF2*, *EZH2*, *IDH1*/*2*, *U2AF1*) and cytogenetic risk, these additional mutations did not retain significant prognostic value [22]. Yan et al. analyzed the mutational landscape in 258 patients with PMF to identify the role of non-driver mutations in disease progression. *ASXL1* mutations were found to be strongly associated with disease advancement and worse prognosis [23]. Mutations in the RAS pathway have also been linked to the PMF phenotype, whereas PV and ET typically harbor subclonal *KRAS*/*NRAS* mutations with low variant allele frequencies (VAFs, <10%). These findings suggest that additional non-driver mutations may affect the phenotype of myeloproliferative neoplasms (MPNs) and contribute to the development of myelofibrosis [24,25].

The presence of HMR mutations is regularly considered for transplant decision making. A recent study evaluated 50 patients with primary and secondary myelofibrosis undergoing hematopoietic stem cell transplant (HSCT) and found the number of HMR mutations is a strong predictor of post-transplant outcomes. Patients with two and more HMR mutations had significantly worse survival and higher non-relapse mortality. Among individual genes, *DNMT3A* and *EZH2* mutations were associated with poor outcomes, and *TP53* mutations predicted higher relapse risk [26].

## 3. Clinical Features of Primary Myelofibrosis

About one fourth to one third of patients with PMF are asymptomatic, and such cases are often discovered during the evaluation of unexplained anemia, splenomegaly, or hepatomegaly.

The classic presenting features of primary myelofibrosis include splenomegaly, teardrop-shaped red blood cells, and leukoerythroblastic changes (appearance of nucleated red blood cells and immature granulocytes) on the blood smear and a hypercellular bone marrow with osteosclerosis and collagen fibrosis. The disease encompasses a wide range of hematologic and systemic manifestations. Anemia is common, and progressive cytopenias, due to declining marrow function, increase the risk of infection and bleeding. Massive splenomegaly may cause abdominal discomfort, early satiety, splenic rupture, portal hypertension, and further cytopenias due to sequestration. Osteosclerosis contributes to bone pain, while advanced stages are marked by severe constitutional symptoms and cachexia. A subset of patients may show thrombocytosis resembling ET. Around 10–20% of patients develop thromboembolic events at diagnosis or over the course of the disease. Extramedullary hematopoiesis can develop in any organ, but more frequently occurs in spleen, liver, vertebral column, and lymph nodes.

## 4. Histopathology of Primary Myelofibrosis

**Figure 1:**
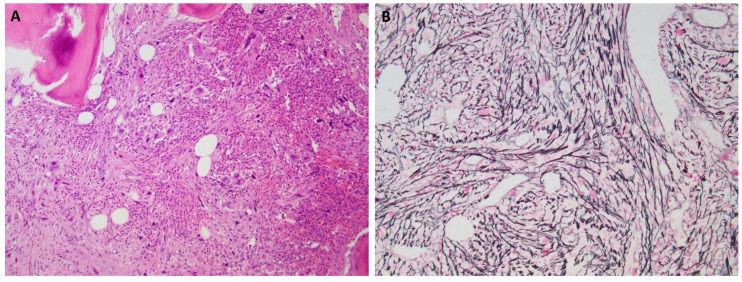
Bone marrow biopsy of primary myelofibrosis, fibrotic stage. The marrow shows megakaryocytic hyperplasia with associated fibrosis. (**A**) The megakaryocytes range in cell size with many large hyperlobulated and hyperchromatic forms (H&E stain, 100×). (**B**) Marked reticulin fibrosis (grade 3 of 3).

The typical histologic feature of PMF is the proliferation of atypical megakaryocytes and myeloid proliferation (Figure 1). Megakaryocytes in PMF display atypical morphology, including prominent clustering, naked nuclei, and hyperchromatic, bulbous (“cloud-like”) nuclei—a feature characteristic of PMF and rarely observed in ET or PV, and need to be distinguished from those in myelodysplastic syndrome. Bone marrow fibrosis is graded between MF-0 (absent) and MF-3 (osteosclerosis with dense collagen fibrosis) on reticulin and trichrome stain [27]. The fibrosis starts with the deposition of reticulin (collagen III) (MF-1), followed by accumulation of collagen I (MF-2/3). Osteosclerosis typically occurs in late-stage PMF and is characterized by bone remodeling and bone formation along with marked fibrosis. Marked bone marrow fibrosis and osteosclerosis often result in dilated sinuses and the presence of intrasinusoidal hematopoiesis in the fibrotic stage of PMF.

Based on the level of bone marrow fibrosis, PMF is further subclassified as prefibrotic PMF (prePMF) or fibrotic stage of PMF. The prePMF is characterized by atypical megakaryocytic and myeloid proliferation and minimal or absent fibrosis (≤MF-1 reticulin fibrosis). The prePMF has a favorable prognosis when compared with the fibrotic stage of PMF and usually lacks advanced splenomegaly or leukoerythroblastosis. The fibrotic stage of PMF is defined by the presence of advanced fibrosis (>1 MF-1 reticulin fibrosis), sometimes increased and sometimes reduced hematopoietic cellularity, and clinical findings like splenomegaly, anemia, and constitutional symptoms. The fibrotic stage PMF has the worst prognosis with a higher rate of AML transformation among *BCR::ABL1* negative MPNs [28,29]. Extramedullary hematopoiesis (EMH), most notably in the liver and spleen, is a consequence of marrow fibrosis displacement of hematopoietic stem cells.

The progressive fibrosis of the bone marrow that characterizes PMF is a consequence of interactions between clonal hematopoietic stem cells (HSCs) and the stromal microenvironment. Fibrosis in PMF is driven by the secretion of profibrotic cytokines, including transforming growth factor-beta (TGF-β), platelet-derived growth factor (PDGF), and vascular endothelial growth factor (VEGF) [30]. Recent studies showed that Gli1+ and Lepr+ mesenchymal stem cells are significant contributors to remodeling of the extracellular matrix (ECM) and fibrosis [31,32]. A number of signaling pathways involved in bone development, such as bone morphogenetic protein (BMP) and canonical Wnt signaling pathway, may cooperate with TGF-β to promote osteosclerosis [33]. The bone marrow frequently shows increased angiogenesis, which is induced by VEGF [34]. The microvessel density correlates with a high *JAK2* V617F allele burden (≥ 55 % mutant alleles) in PMF [35].

## 5. Cytogenetic Findings of Primary Myelofibrosis

Karyotypic abnormalities can be seen in up to 45% of PMF cases. The most common recurrent karyotypic abnormalities include del(20q), del(13q), trisomy 8, and trisomy 9 [36]. Patients with favorable karyotypes, such as normal karyotype, 20q-, 13q-, trisomy 9, and Y-, expect a median OS of 4.4 years. Unfavorable karyotypes, including +8, −5/5q-, 7q-, complex karyotypes, have a median OS of 2.9 years. Very high-risk (VHR) karyotypes, such as −7, inv(3)/3q21, i(17q), 12p-, 11q-, and trisomy’s other than +8 or +9, are associated with a median OS of 1.2 years [36]. It is of note that these OS numbers are lower than the median overall survival of 9 years in the general PMF patients as reported in the previously described study [3], which might include more patients with only normal karyotype.

## 6. Diagnostic Criteria of Primary Myelofibrosis

WHO-5th and the 2022 ICC provide defined diagnostic criteria of PMF. Diagnosis of prePMF or overt PMF requires all three major criteria and at least one minor criterion on two consecutive assessments.

The first major criterion differs between the prefibrotic and overtly fibrotic forms. In prePMF, it includes megakaryocytic proliferation and atypia on bone marrow biopsy, increased age-adjusted cellularity, granulocytic proliferation, often with decreased erythropoiesis, and bone marrow fibrosis < grade 2. In fibrotic PMF, it is defined by reticulin and/or collagen fibrosis of grade 2 or 3. The other two major criteria are the same for both forms: (2) presence of a clonal marker such as *JAK2*, *CALR*, or *MPL* mutation, or another clonal abnormality, and (3) exclusion of other myeloid neoplasms, including *BCR::ABL1*-positive chronic myeloid leukemia, PV, ET, myelodysplastic syndromes, or other myeloid disorders.

The minor criteria include the following: (1) anemia not explained by comorbid conditions, (2) leukocytosis ≥11 × 10^9^/L, (3) palpable splenomegaly, and (4) elevated lactate dehydrogenase. Fibrotic PMF also includes leukoerythroblastosis as an additional minor criterion.

Prefibrotic PMF has a more favorable prognosis than fibrotic PMF and can be mis-diagnosed as ET or PV or other MPNs. It is associated with higher thrombotic risk than ET [37]. Its accurate diagnosis requires the integration of clinical presentation, lab results, molecular and genetic findings, and morphological features. Its management focuses on reducing symptoms and thromboembolic events and preventing disease progression. JAK2 inhibitors like ruxolitinib have shown some effects but their role in prePMF is still under investigation.

PMF can progress to an accelerated phase (AP), characterized by a blast count of 10–19% in the peripheral blood or bone marrow, often forming clusters of CD34-positive blasts detectable by immunohistochemistry. Transformation to the blast phase (BP), or leukemic transformation, is defined by a blast count of ≥20%, consistent with acute myeloid leukemia. Table 2 outlines the PMF diagnostic criteria as defined by the WHO-5th and the ICC.

## 7. Comparison and Differentiation with Other Myeloid Neoplasms

### 7.1. Essential Thrombocythemia (ET)

PrePMF can mimic ET clinically and morphologically with presentation of thrombocytosis and megakaryocyte proliferation. It is important to differentiate prePMF from ET as they have significant difference in prognosis [38]. Patients with prePMF have a significantly worse clinical course, with lower overall survival and increased risk of progression to AML and the fibrotic stage of the disease. In contrast to patients with ET, patients with prePMF are more likely to have leukocytosis, higher LDH value, higher number of circulating CD34-positive blasts, and more frequent splenomegaly. Careful evaluation of morphologic features usually can distinguish prePMF from ET. The megakaryocytes in ET are relatively uniform in size with large hyperlobulated “staghorn-like” megakaryocytes but without significant hyperchromatic. They may form loose clusters but usually are more evenly distributed in the marrow. In contrast, the megakaryocytes in the prePMF show more prominent clustering, abnormal paratrabecular location, and variable cell sizes, ranging from small to large, hyperlobulated forms with frequent hyperchromatic nuclei. Megakaryocytes with dysmaturation, including the typical “bulbous” nuclei, are highly specific for prePMF. These morphologic features of megakaryocytes along with peripheral blood leukocytosis and increased LDH level as defined in the WHO classification as minor criteria can help establish the diagnosis of prePMF and rule out ET.

Genetic findings can help differentiate prePMF from ET. *JAK2*^V617F^ allele burden can help discriminate ET from prePMF in a subset of cases [39], as approximately a quarter of prePMF patients have *JAK2*^V617F^ allele burden of more than 50%, while ET patients show lower *JAK2*^V617F^ allele burden of less than 40%. Overall, prePMF has more frequent *CALR* mutations than ET (35·8% vs. 17·8%) [38]. Type 1-like *CALR* mutations are significantly more frequent in PMF, while type 2-like mutations are more common in ET [14,40]. *MPL* W515L/K mutations are more frequently identified in prePMF than ET (5% vs. 1%) [41].

### 7.2. Post-Polycythemia Vera Myelofibrosis and Post-Essential Thrombocythemia Myelofibrosis

Both PV and ET can develop into fibrotic stage of the disease as post-PV myelofibrosis (PPV-MF) and post-ET myelofibrosis (PET-MF) with disease progression, usually years or decades after initial diagnosis. Both PPV-MF and PET-MF cannot be reliably differentiated from the fibrotic stage of PMF both clinically and morphologically. Patients with PPV-MF and PET-MF usually show increasing splenomegaly, anemia, and leukoerythroblastosis in the peripheral blood. Their bone marrow shows abnormal large megakaryocytes with more prominent clustering, hyperlobulation, and frequent hyperchromatic nuclei, as well as grade 2–3 reticulin fibrosis. The differential diagnosis is clinically relevant, as the median overall survival is longer in patients with PET-MF (73 months) versus PMF (45 months) and PPV-MF (48 months) [42]. The diagnosis of PPV-MF and PET-MF can only be confirmed by documented history of prior diagnosis of PV or ET. The identification of *JAK2* exon 12 mutations can confirm the diagnosis of PPV-MF, as *JAK2* exon 12 mutations are exclusively seen in PV. Table 3 summarizes the major features of PMF, PET-MF, and PPV-MF.

### 7.3. Chronic Myeloid Leukemia (CML)

CML can mimic either the early (prefibrotic) phase of primary myelofibrosis (PMF)—particularly when presenting with thrombocytosis or the fibrotic phase of PMF, due to overlapping features such as bone marrow fibrosis and splenomegaly. Notably, bone marrow fibrosis may occur in approximately 40% of cases of CML and is associated with a poor prognosis, though it may be reversible via tyrosine-kinase inhibitors (TKIs) [43]. Despite these similarities, PMF can be easily distinguished from CML by morphology and genetic profile (lack of BCR-ABL). CML usually shows basophilia and the bone marrow shows characteristic hypolobulated “dwarf” megakaryocytes, in contrast to the large hyperlobulated hyperchromatic megakaryocytes and abnormal megakaryocytes with “bulbous” nuclei in PMF. The identification of *BCR::ABL1* fusion by PCR or FISH is the hallmark for CML diagnosis.

### 7.4. Myelodysplastic Neoplasms and Myelodysplastic/Myeloproliferative Neoplasms

**Figure 2:**
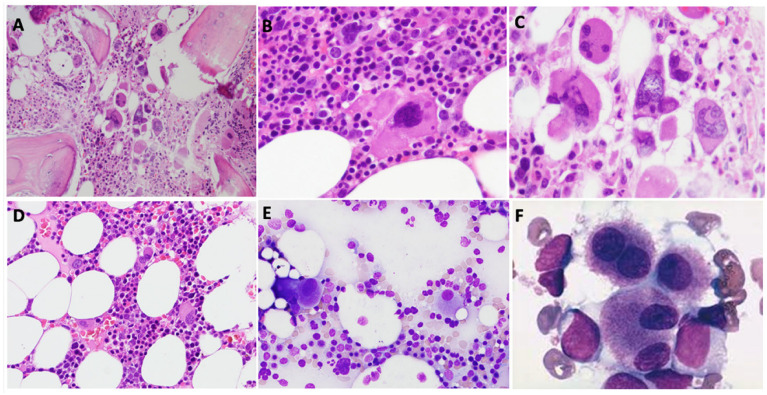
Comparison of the typical megakaryocyte morphology of PMF (**A**–**C**) and MDS (**D**–**F**). The megakaryocytes in PMF are usually increased in number and form dense clusters (**A**), and show hyperchromatic (**B**), bulbous or cloud-like morphology (**C**); while the megakaryocytes in MDS usually do not form dense clusters (**D**), and show mostly small monolobated dysplastic forms (**E**) or nuclear separation (**F**).

Myelodysplastic neoplasm with fibrosis (MDS-F) mimics PMF with anemia and fibrosis [44,45]. MDS-F shows increased blasts and clinically presents with cytopenia and no leukocytosis or thrombocytosis as usually seen in PMF. The megakaryocytes in MDS-F differ from that of PMF with predominance of small, hypolobulated megakaryocytes or micro-megakaryocytes (Figure 2). The mutation profile of MDS-F is distinct from that of PMF. MDS-F usually show mutations in *ASXL1*, *SF3B1*, *TET2*, *RUNX1*, etc., and no *JAK2*, *CALR*, *MPL* mutations.

Chronic myelomonocytic leukemia (CMML) can also mimic PMF with fibrosis [46]. Bone marrow fibrosis is associated with shorter progression-free survival, splenomegaly, and increased megakaryocytes. On the other side, monocytosis can develop in patients with PMF during disease progression. This differential diagnosis can usually be resolved by reviewing the morphology of the megakaryocytes and mutation profiles. CMML typically shows MDS-type megakaryocytes with small, hypolobulated forms and micro-megakaryocytes. CMML shows characteristic mutations in *ASXL1*, *TET2*, *SRSF2,* and *RAS* pathway genes, and no MPN driver mutations. Of note, there are some cases with overlapping features between CMML and PMF, which show co-mutations involving *JAK2* or *MPL* and *ASXL1*, *SRSF2*, *TET2*, *NRAS*, and/or *KRAS* [47]. These myeloid neoplasms may represent a true gray zone between CMML and PMF.

Myelodysplastic/Myeloproliferative Neoplasm with Ring Sideroblasts and Thrombocytosis (MDS/MPN-RS-T) can also share clinical (leukocytosis and thrombocytosis) and pathological (*JAK2* mutation, hyperlobated megakaryocytes, fibrosis) with PMF. However, patients with PMF are more likely to have splenomegaly, *CALR* or *MPL* mutations, and megakaryocytes with hyperchromatic nuclei, while patients with MDS/MPN-RS-T tend to have SF3B1 mutations, a much more increased number of ring-sideroblasts, and multilineage dysplasia.

### 7.5. Systemic Mastocytosis (SM)

SM frequently shows fibrosis associated with peri-trabecular and paratrabecular aggregates of mast cells [48]. The diagnosis is straightforward with identification of aggregates or nodules of neoplastic mast cells with abnormal expression of CD2, CD25 and/or CD30, and *KIT* D816V by molecular methods. It is important to evaluate megakaryocyte morphology in SM. Presence of large, hyperlobated and/or hyperchromatic megakaryocytes should prompt evaluation of the presence of *JAK2*, *CALR* or *MPL* mutations by NGS or PCR, as PMF may coexist with SM as an associated hematological neoplasm (SM-AHN) [49].

### 7.6. Metastatic Carcinoma

Bone is a frequent site of involvement for a variety of metastatic carcinomas. Metastatic carcinomas like prostate carcinoma and breast cancer frequently induce fibrosis in the marrow [50]. Fibrosis in such cases may be extensive and mask the presence of metastatic carcinoma and mimic the fibrotic stage of PMF. Immunohistochemical staining for cytokeratins should be included in the panel of stains in such cases and will confirm the diagnosis of metastatic carcinoma.

### 7.7. Autoimmune Myelofibrosis (AIMF)

AIMF can mimic PMF with fibrosis, leukoerythroblastosis, splenomegaly, and megakaryocyte atypia in a subset of cases [51,52]. AIMF occurs in the presence or the absence of systemic autoimmune disease. Patients typically exhibit cytopenias and bone marrow fibrosis, with the marrow often hypercellular and containing benign lymphoid aggregates and occasionally large, abnormally lobulated or hyperchromatic megakaryocytes. Unlike PMF, AIMF is usually associated with autoimmune antibodies, such as anti-nuclear, anti-double stranded DNA, anti-phospholipid antibody/lupus anticoagulant antibodies and rheumatoid factor, and no driver mutations in *JAK2*, *CALR*, *MPL*.

### 7.8. Inflammatory and Infectious Causes

Infections including HIV and tuberculosis or granulomatous inflammation may induce bone marrow fibrosis [53]. These conditions lack the atypical megakaryocytes characteristic of PMF. Lab tests, such as serology, PCR, and special stains on the tissue (for example, Acid-Fast Bacilli stain for Tuberculosis) are necessary for the diagnosis.

## 8. Conclusions

The area of PMF research has come a long way with integrated WHO/ICC classification criteria, which facilitate the application of prognostic models and the stratification of patients with PMF for optimal treatment. Morphologic and molecular markers by NGS help to distinguish PMF from other MPNs, MDS, and reactive processes, while molecular markers (e.g., *ASXL1*, *SRSF2*, *TP53*) can additionally guide individualized therapy.

JAK inhibitors, approved for intermediate-2 and high-risk MF, are effective across all subgroups, including TN-PMF. In COMFORT-I, 59% achieved ≥35% spleen volume reduction regardless of JAK2 status; COMFORT-II reported a 28% reduction at 48 weeks with symptoms and quality-of-life improvements regardless of mutational status. These results highlight the central role of JAK-STAT dysregulation in MF pathogenesis. However, JAK inhibitors do not significantly alter disease progression or survival.

Future research should focus on disease-modifying therapies to improve outcomes across all MF subtypes, including TN-PMF [54].

## Figures and Tables

**Table 1:** Major laboratory and morphologic findings and driver gene mutations in PML, PV and ET.

	Laboratory and Morphologic Findings	Driver Mutations
Primary myelofibrosis	Leukocytosis Hyperlobulated and Hyperchromatic megakaryocytes, megakaryocytes with “bulbous” nuclei	*JAK2* V617F ~60% *CALR* ~25–30% *MPL* ~5% Triple negative ~5–10%
Essential thrombocythemia	Thrombocytosis Hyperlobulated megakaryocytes with “Staghorn-like” nuclei	*JAK2* V617F ~60% *CALR* ~20–25% *MPL* ~5% Triple negative ~10%
Polycythemia Vera	Erythrocytosis Panmyelosis with large hyperlobulated nuclei Decreased erythropoietin	*JAK2* V617F ~96% *JAK2* exon 12 mutation ~4%

**Table 2:** WHO-5th and ICC diagnostic criteria for PMF.

PMF, Prefibrotic/Early Stage (Requires All 3 Major Criteria and at Least 1 Minor Criterion)	
**Major Criteria**	
	Megakaryocytic proliferation and atypia, without reticulin fibrosis grade > 1, accompanied by increased age-adjusted bone marrow cellularity, granulocytic proliferation, and (often) decreased erythropoiesis WHO criteria for BCR-ABL1-positive CML, PV, ET, MDS, or other myeloid neoplasms are not met JAK2, CALR, or MPL mutation; or presence of another clonal marker (e.g., ASXL1, EZH2, TET2, IDH1, IDH2, SRSF2, and SF3B1 mutations); or absence of minor reactive bone marrow reticulin fibrosis
**Minor Criteria** (At least one, confirmed in 2 consecutive determinations)	
	Anemia not attributed to a comorbid condition Leukocytosis ≥ 11 × 10^9^/L Palpable splenomegaly Lactate dehydrogenase level above the upper limit of the institutional reference range
**PMF, Overt Fibrotic Stage** (requires all 3 major criteria and at least 1 minor criterion) **Major Criteria**	
	Megakaryocytic proliferation and atypia, accompanied by reticulin and/or collagen fibrosis grades 2 or 3 WHO criteria for BCR-ABL1-positive CML, PV, ET, MDS, or other myeloid neoplasms are not met JAK2, CALR, or MPL mutation; or presence of another clonal marker (e.g., ASXL1, EZH2, TET2, IDH1, IDH2, SRSF2, and SF3B1 mutations); or absence of reactive fibrosis
**Minor Criteria** (At least one, confirmed in 2 consecutive determinations)	
	Anemia not attributed to a comorbid condition Leukocytosis ≥ 11 × 10^9^/L Palpable splenomegaly Lactate dehydrogenase level above the upper limit of the institutional reference range Leukoerythroblastosis
**AP**(10–19% blasts) **and BP **(≥20% blasts)	

**Table 3:** Differential diagnosis of PMF, post-polycythemia vera myelofibrosis, and post-essential thrombocythemia myelofibrosis.

Diagnosis	Prefibrotic Primary Myelofibrosis	Fibrotic Primary Myelofibrosis	Post-Essential Thrombocythemia Myelofibrosis	Post-Polycythemia Vera Myelofibrosis
Bone marrow	Megakaryocytic proliferation and atypia			
	Increased age-adjusted BM cellularity, granulocytic proliferation, reticulin fibrosis < 2	Reticulin and/or collagen grade 2 or 3		
			Previously established diagnosis of ET	Previously established diagnosis of PV
Laboratory and clinical findings	Anemia Palpable splenomegaly LDH level above the range			
	Leukocytosis ≥ 11 × 10^9^/L	Leukoerythroblastosis Constitution symptoms		
	*JAK2 V617F*, *CALR,* and *MPL*			*JAK2 V617F*, *JAK2* Exon 12

## Data Availability

No new data were created.
